# Regional variations of child development index in Bangladesh

**DOI:** 10.1016/j.heliyon.2021.e07140

**Published:** 2021-05-27

**Authors:** Md. Ismail Hossain, Iqramul Haq, Maliha Afroj Zinnia, Mafruha Sultana Mila, Md. Iqbal Hossain Nayan

**Affiliations:** a Department of Statistics, Jagannath University, Dhaka 1100, Bangladesh; b Department of Agricultural Statistics, Sher-e-Bangla Agricultural University, Dhaka 1207, Bangladesh; c Department of Pharmacy, East West University, Dhaka 1212, Bangladesh; d Research & Development Department, Orion Pharma Ltd, Bangladesh

**Keywords:** Child development, Early school attend, Cognitive development, Nutrition

## Abstract

Early development is a vital phase in childhood life. The study aimed to identify factors that were associated with the early development of 36–59 months children in Bangladesh. The findings of this study will formulate the design of appropriate policy and programmed responses.

Utilizing Multiple Indicator Cluster Survey data, influencing components of child development status were evaluated for both rural and urban areas of Bangladesh. A total of 23,099 children under the age of five were included in this analysis. Chi-square analysis was conducted to assess the association between outcome variables and selected covariates. At the same time, this study uses two separate multivariate binary logistics regression models (respectively for urban areas and rural areas) to determine the risk factors that are primarily related to child development.

Our research estimates that more than 70 percent of children develop early throughout the country. The multivariate analysis on the determinants of child development index among children aged between 36 and 59 months old regarding residence discovered a significant impact on child age and sex, maternal education, child education, wealth status, reading children's books. The adjusted odds of child nutrition status, playthings, and maternal functional difficulties have had a major impact on early child development in rural Bangladesh.

Based on the findings, educational status, nutritional status, wealth-status, and some determinants of children care the most noteworthy findings in this study. Hence, policymakers should emphasize on such factors for improving children's development in residence.

## 1 Introduction

The development of children is the primary determinant that affects the health of children throughout their lives [1]. Early childhood development (ECD) comprises physical, socio-emotional, cognitive, and motor growth between 0 and 8 years of age [2, 3, 4]. Early Child Development is also known by many terms, such as Early Childhood Care and Development, Early Childhood Education, Early Childhood Care and Education etc. [5, 6]. Early-stage healthy development is the building blocks of individual's educational achievement, good wellbeing, stable societies, and effective parenting as well as for societies stability and economic growth [7].

Two Lancet series, published in 2007 and 2011, were conducted on child development for developing countries. These articles reported that in low and middle-income countries around 219 million children (39 percent) under five years of age are at threat not to reach their developmental potential. In these countries, children lack adequate earning skills as they grow up, resulting in an average annual deficit of adult income of 19.8 percent which attracts worldwide attention [8, 9]. A study of developing countries found four fundamental factors like individual child activity and wellbeing, family life, learning space, and socio-economic condition which impacted the development of children [10]. Again, the field had steadily grown as the Lancet predicted a series of studies between 2004 and 2010 and proclaimed that the approximate number of children under the age of five in LMICs was threatened by stunting or severe poverty [11, 12]. South Asia had the highest decline in underdeveloped risk children, from 110.9 million in 2004 to 88.8 million in 2010. In contrast, children in sub-Saharan Africa had the highest prevalence of unmet growth potential (70 percent in 2004 and 66 percent in 2010) [12].

Early childhood development programs in Bangladesh differ in duration and intensity and primarily focused on pre-school children [13, 14]. Bangladesh has future attention in Pre-Primary Education (PPE) provision, which was to establish a minimum standard of quality in all schools and concentrate on sectors where education disparities are most severe [4, 15]. To ensure children's future accomplishments, enhancing the early years of their lives will be the best investment for society [16].

UNESCO, UNICEF, the World Bank, and various agencies are committed to promoting early childhood development and the World Health Organization's dedication to lead a Global Strategy for Women's, Children's and Adolescents Health 2016–2030 [17,18]. Early child development is increasingly recognized as an important global growth indicator, including the Sustainable Development Goals (goal 4.2.1) [19,20]. Though the data on early child development in low middle-income countries is still limited, the United Nations Children's Fund (UNICEF) recently started collecting data on the Early Childhood Development Index (ECDI), which can assess four development areas: literacy, physical, social-emotional, and learning [21].

This study was designed to examine factors associated with early child development among children aged between 3 and 5 years in both urban and rural areas of Bangladesh.

## 2 Methods and materials

### 2.1 Source of data

This study analyzed secondary data from the Bangladesh Multiple Indicator Cluster Survey (MICS), 2019. Bangladesh MICS is a nationally representative survey managed by the Bangladesh Bureau of Statistics (BBS) with the technical support and fund of UNICEF [22]. The MICS collects and provides a wide range of information on mother and child health, child development under five years, and household characteristics in all divisions and districts in Bangladesh.

### 2.2 Sample design and sample size

**Figure 1:**
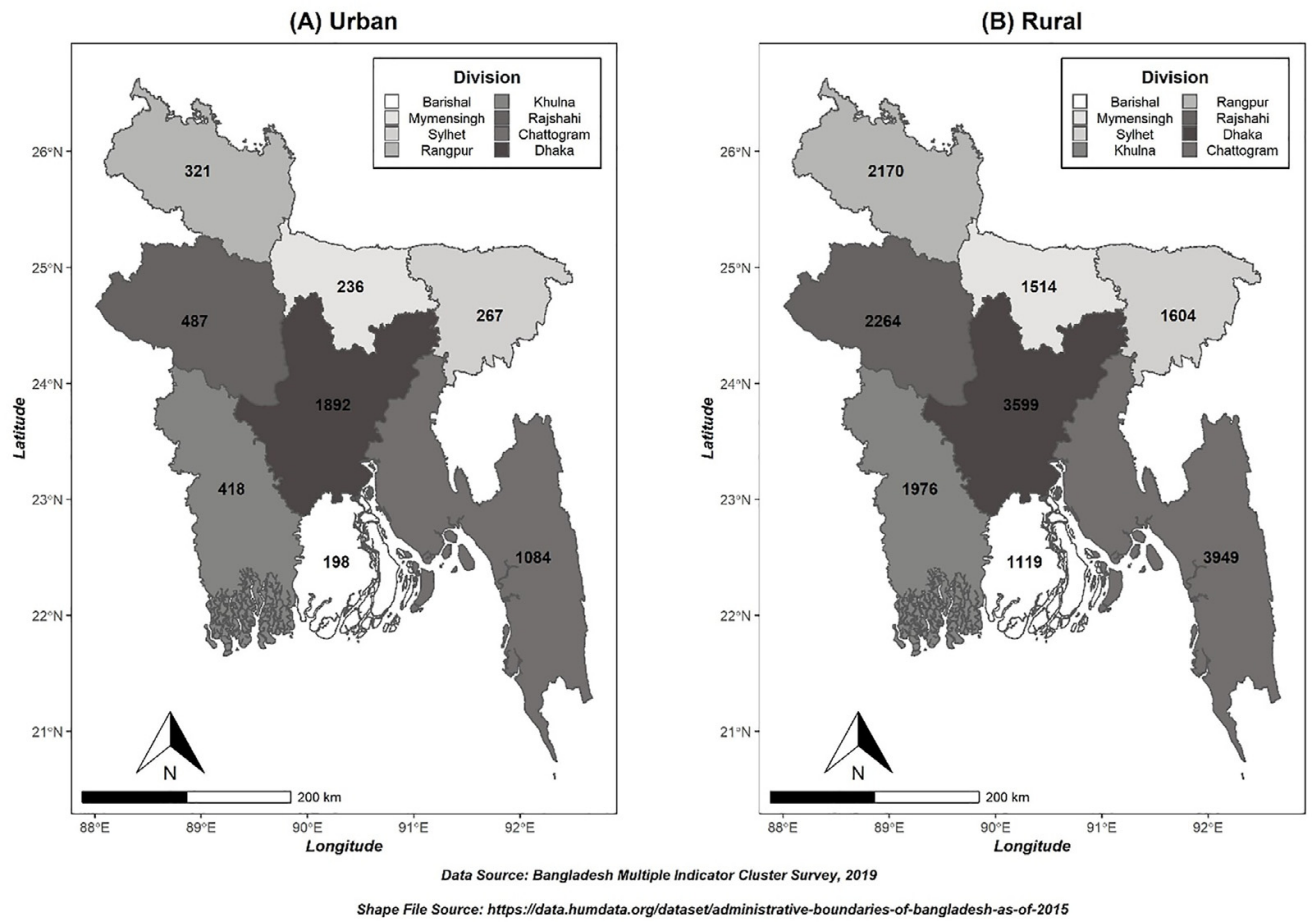
Map of sample's geographical locations in (A) Urban and (B) Rural areas of Bangladesh.

The MICS provides information on children under five years old at district, division, and national levels of Bangladesh. A two-stage stratified cluster sampling method was used for sample selection and used a similar sampling frame, which was also used in the 2011 Bangladesh Population and Housing Census [23]. At the time of data collection, there were 64 districts which were defined as the sampling cluster, and 3220 enumeration areas (EAs) were defined for the census enumeration, which was the primary sampling unit. In the second stage, a systematic sample of 20 households (on average) was drawn from each sample EAs. The Bangladesh Multiple Indicator Cluster Survey comprised 64,400 households from the country as a whole, and 24,686 were eligible for the interview. After weighting, the final sample size of this study appeared to be 23,099 mothers/caretakers with children under the age of five in Bangladesh, with 4,903 mothers/caretakers with children under the age of five were from urban areas and 18,196 mothers/caretakers with children under the age of five were from rural areas of Bangladesh (see Figure 1).

### 2.3 Dependent variable

The Early Child Development Index (ECDI) characterized the percentage of children aged from 36 to 59 months old and measured using four domains, namely Literacy-numeracy, Physical, Social-emotional, and Learning. Table 1 contains 10 item modules used to determine these domains.

**Table 1:** The module of early child development index.

Module Number	Module Description	Track if the answer is
Module 1	Child identifies at least ten letters of the alphabet.	Yes
Module 2	Child reads at least four simple, popular words.	Yes
Module 3	Child knows name and recognizes symbol of all numbers from 1-10.	Yes
Module 4	Child able to pick up small/tinny object (a stick/rock) from ground with two fingers	Yes
Module 5	Child sometimes too sick to play.	No
Module 6	Child gets along well with other children.	Yes
Module 7	Child kicks, bites or hits other children or adults.	No
Module 8	Child gets distracted/diverted easily.	No
Module 9	Child follows simple directions.	Yes
Module 10	Child able to do something independently.	No

When a child is being monitored to have at least two modules from modules 1,2 and 3 then he/she can be distinguished to be developmentally on track in the literacy-numeracy domain. Developmentally on track in the physical domain is considered from modules 4 and 5 if at least one item is observed to be on track. The child is considered as developmentally on track in the social-emotional domain if two items are accurate from modules 6,7 and 8. And also, if a child meets at least one item on track between modules 9 and 10 then we considered him or her as being developmentally on track in the learning domain.

For pointing out domain compliance for each child i, aged of 36–59 months old in Bangladesh for the domain r (literacy-numeracy, physical, social-emotional, learning),

(1) $$d_{i,r}=\left\{ \begin{array}{l} 1;\;if\;child\;i\;in\;Bangladesh\;is\;on\;track\;in\;domain\;r\\ 0;\;if\;child\;i\;in\;Bangladesh\;is\;not\;on\;track\;in\;domain\;r \end{array}\right.$$

analyzed Early Child Development (ECD) compliance for each child i, aged 36–59 months, in Bangladesh, assures at least 3 out of 4 domains.

That is,

(2) $$ECD_{i}=\left\{ \begin{array}{l} 1;\;if\;\overset{4}{\underset{r=1}{\sum }}d_{i,r}\geq 3\\ 0;\;Otherswise \end{array}\right.$$

Furthermore, the Early Child Development Index (ECDI) was the percentage of children who were developmentally on track in at least three out of these four domains.

(3) $$ECDI=\left\{ \begin{array}{l} 1;\;Yes,\;developmentally\;on\;track\;in\;at\;least\;three\;domains\;\\ 0;\;No,\;not\;developmentally\;on\;track\;in\;at\;least\;three\;domains \end{array}\right.\;$$

The created binary variable was considered as the dependent variable for the final analyses.

### 2.4 Independent variable

A set of socio-demographic risk factors associated with the early child development status of children were considered as covariates. In this study, Child current age in months (36–47 months, 48–59 months), Child sex (Male and Female), Mother's education level (No education, Primary education, Above the secondary level of education), Wealth status (Poor, Middle, Rich), Attendance to early childhood education (Attending, Not attending), Child nutrition status (Severely malnourished, Moderately malnourished, Nourished), Reading three or more children's books (Yes, No), Child have playthings (Yes, No), Functional difficulties for mother (Has functional difficulties, Has no functional difficulties) were selected as independent variables for this analysis.

### 2.5 Analytical procedure

Reasonable statistical methods were adopted for analyzing the data to attain various objectives of this study. Bivariate analysis was used to create interconnections between socio-demographic variables, and dependent variables. In the bivariate framework, the chi-square test of independence has been applied to assess the association between ECDI with selected covariates. Mathematically, the chi-square statistics can be defined as,

(4) $$\chi^{2}=\overset{n}{\underset{i=1}{\sum }}\frac{{\left(Observed\;frequency_{i}-Expected\;frequency_{i}\right)}^{2}}{Expected\;frequency_{i}}\;$$

This statistic follows a chi-square distribution with (Numberofrow−1)×(Numberofcolumn−1) degrees of freedom.

In a multivariate setting, a popular multivariate model called the Binary Logistic Regression model [24] can predict binary categorical dependent variables based on continuous or categorical independent variables to determine the influence of independent variables on dependent variables. As we all know, the Binary Logistic Regression model was more effective and accurate in analyzing binary data. The odds ratio with a 95% confidence interval was usually used to explain predictor variables impact.

Let $D_{i}$ denote the binary dependent variable for the $i^{th}$ observation.

(5) $$\text{Where,}\;D_{i}=\left\{ \begin{array}{l} 1,\;Yes,\;developmentally\;on\;track\;in\;at\;least\;three\;domains\;\\ 0,\;No,\;not\;developmentally\;on\;track\;in\;at\;least\;three\;domains \end{array}\right.\;$$

$I_{i1},I_{i2},\ldots ,I_{ip}$ be a set of independent variables which can be quantitative or indicator variables referring to the level of categorical variables.

Since D was a binary variable, it has a Bernoulli distribution with parameter $\pi_{i}$. The dependence of the probability of success on independent variables is assumed to be respectively as-

(6) $$P\left(D_{i}=1\right)=\pi_{i}=\frac{\text{exp}\left(\beta_{0}+\beta_{1}I_{i1}+\ldots +\beta_{p}I_{ip}\right)}{1+\text{exp}\left(\beta_{0}+\beta_{1}I_{i1}+\ldots +\beta_{p}I_{ip}\right)}$$

The above relation also can be expressed as,

(7) $$g\left(I\right)=logit\left(\pi_{i}\right)=log\frac{\pi_{i}}{1-\pi_{i}}=\beta_{0}+\beta_{1}I_{i1}+\ldots +\beta_{p}I_{ip}$$

The likelihood is maximized by finding estimates of the parameters that are most likely to give us the data. The maximum likelihood estimator (MLE) of $\beta_{0}$ and $\beta_{1}$ can be obtained by maximizing:

(8) $$L\left(\beta_{0},\beta_{1}\right)=\overset{n}{\underset{i=1}{\prod }}\frac{\text{exp}\left\{D_{i}\left(\beta_{0}+\beta_{1}I_{i}\right)\right\}}{1+\text{exp}\left(\beta_{0}+\beta_{1}I_{i}\right)}$$

All statistical analyses were performed using SPSS version 25 (IBM, Corporation, Armonk, NY, USA) and R version 3.6.0 (Bell Laboratories, New Jersey, USA).

## 3 Results

### 3.1 Geographical conception of early child development index

**Figure 2:**
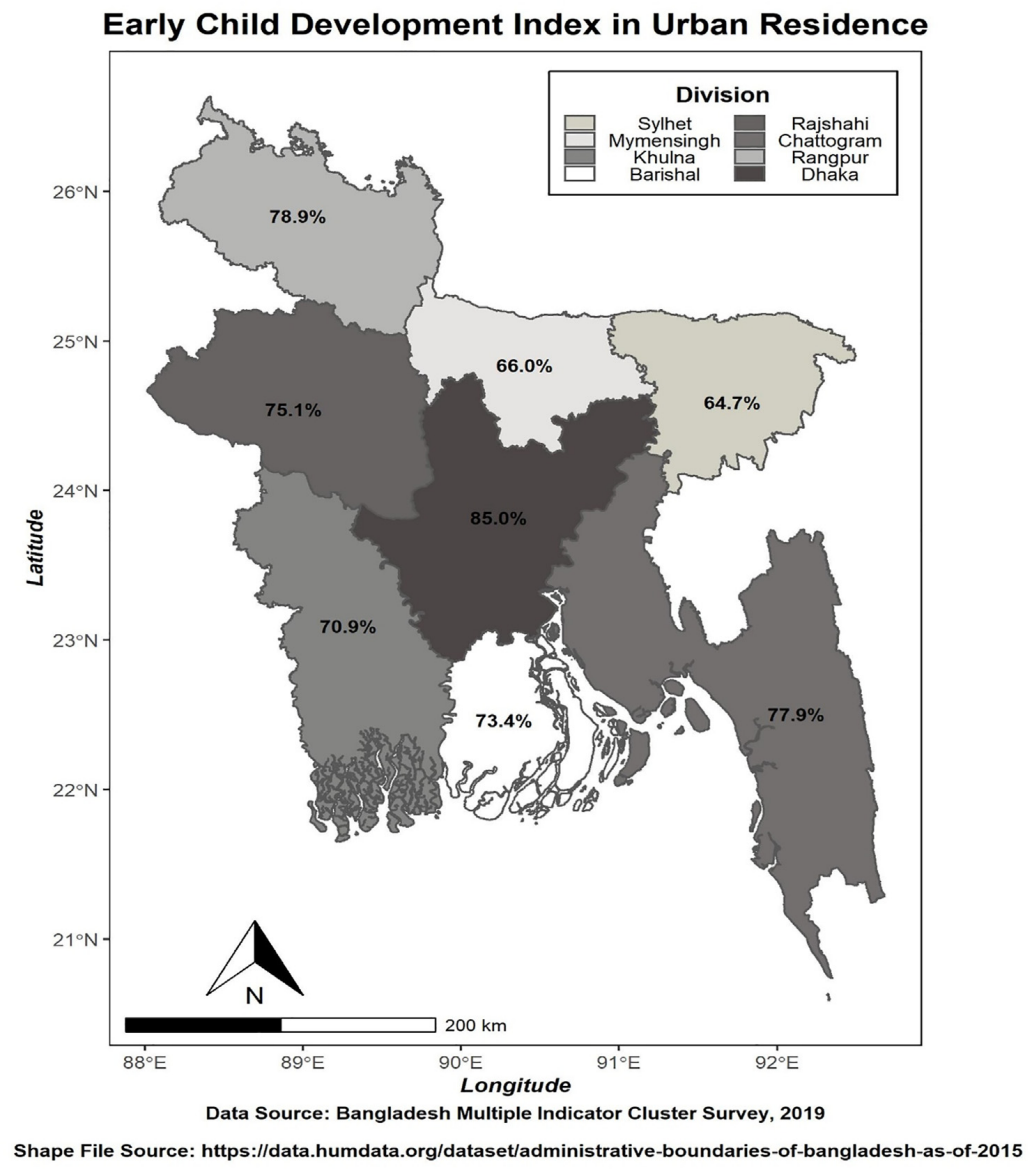
Spatial illustration of Early Child Development Index in the Urban residence of Bangladesh.

**Figure 3:**
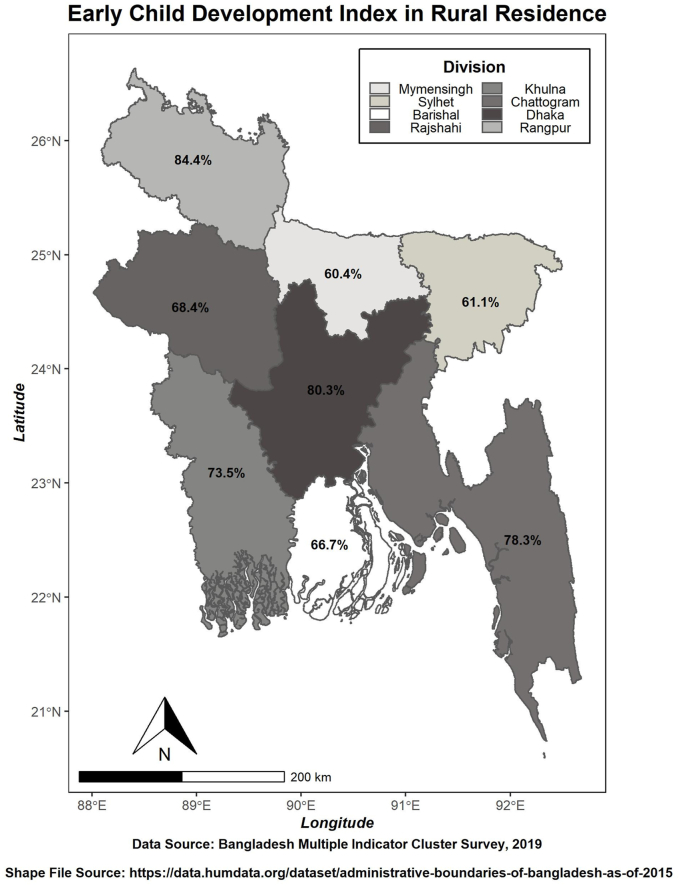
Spatial illustration of Early Child Development Index in the Rural residence of Bangladesh.

About 79 percent of children in Bangladesh fulfilled at least three domains before their fifth birthday. The following maps (Figure 2 and Figure 3) showed the urban and rural Early Child Development Index (ECDI) for Bangladesh in terms of division. Concerning the urban residence (Figure 2), the Dhaka division (85 percent of children had developed at least three domains at the age between three and five years) demonstrated the highest score, while the Sylhet division possessed the lowest index score (64.7 percent). Correspondingly, the Rangpur division had the most significant index score (approximately 84 percent of children had developed at least three domains). The Mymensingh division possessed the lowest (60.4 percent) regarding rural residence (Figure 3). Division occupying the highest ECDI indicates that the children below five years in that area had completed at least three domains. However, more surveillance is needed in the lowest ECDI area for improvement.

### 3.2 Association between socio-demographic factors on early childhood development index

Table 2 clarified the association between the percentage distribution and early childhood development index of 36–59 months old children, which was accompanied by the socio-demographic characteristics in both urban and rural areas of Bangladesh. It also represented the factors significantly associated (p < 0.001 or p < 0.01) with early childhood development index contained child age and sex, mother's education, early childhood education, wealth status, reading children's books, having playthings for both residential areas in the survey in Bangladesh.

**Table 2:** Cross-tabulation analysis of Early Childhood Development Track among children by different socio-demographic characteristics in Bangladesh.

Background Characteristics	Developmentally on track in at least three domains			
	Urban		Rural	
	Yes (%)	No (%)	Yes (%)	No (%)
**Child Age**				
36–47 Months	72.3	27.7	68.0	32.0
48–59 Months	84.3	15.7	80.4	19.6
**p-value**	p < 0.001		p < 0.001	

**Child Sex**				
Male	74.8	25.2	70.6	29.4
Female	81.8	18.2	77.6	22.4
**p-value**	p < 0.001		p < 0.001	

**Mother's Education**				
No Education	69.2	30.8	68.4	31.6
Primary Education	69.9	30.1	69.3	30.7
Secondary and above	82.0	18.0	77.2	22.8
**p-value**	p < 0.001		p < 0.001	

**Early Childhood Education**				
Attending	89.4	10.6	84.9	15.1
Not attending	74.7	25.3	71.6	28.4
**p-value**	p < 0.001		p < 0.001	

**Wealth Status**				
Poor	67.7	32.3	70.0	30.0
Middle	70.4	29.6	76.1	23.9
Rich	81.2	18.8	79.2	20.8
**p-value**	p < 0.001		p < 0.001	

**Children Nutrition Status**				
Severe Malnourished	76.0	24.0	66.6	33.4
Moderate Malnourished	78.2	21.8	71.6	28.4
Nourished	78.4	21.6	75.3	24.7
**p-value**	p = 0.918		p < 0.001	

**Reading Children's Books**				
≥3 books	87.8	12.2%	84.6	15.4
<3 books	75.6	24.4	72.8	27.2
**p-value**	p < 0.001		p < 0.001	

**Having Playthings**				
Yes	78.8	21.2	74.9	25.1
No	67.9	32.1	66.2	33.8
**p-value**	p < 0.01		p < 0.001	

**Mother's Functional Difficulties**				
Has functional difficulty	78.1	21.9	60.2	39.8
Has no functional difficulty	78.1	21.9	74.2	25.8
**p-value**	p = 0.597		p = 0.001	

Statistical Significance: ∗p < 0.05, ∗∗p < 0.01, ∗∗∗p < 0.001.

Child age is significantly and positively interconnected with early child development for both areas (urban and rural) of Bangladesh, where almost 85 percent of children of 48–59 months old were shown to be developed in at least three domains. In terms of child sex, around 82 percent of female children in urban areas and 78 percent of female children in rural areas developed early in at least three domains, which indicated that urban children were more likely to developed than rural children. From Table 2, it was prominent that male children were less likely to be developed than female children (74.8 percent in an urban area and 70.6 percent in a rural area).

Moreover, a significant positive association between a mother's education and early childhood development was revealed by our data in both areas of Bangladesh, as mothers holding secondary and above education displayed 82 percent and 77.2 percent children development on track in at least three domains for urban and rural areas respectively. Again, the effect of early childhood education on children's development was showed in Table 2, where almost 89.4 percent of children in urban areas and 85 percent of children in rural areas were noted to be developed early if attained early childhood education. Children from rich family's experienced about 81.2 percent early development whereas 79.2 percent of children's development occurred in urban and rural areas successively, which demonstrated the direct correlation of the wealth status and early childhood development. On top of that, severely malnourished children tend to experience lesser developmental status in at least three domains (76 percent in urban area and 66.6 percent in rural area) than the children who were nourished or moderately malnourished.

Furthermore, two factors were also observed to be associated with early childhood development. Firstly, reading books (three or more) influenced on the early development of children (87.8 percent in urban and 84.6 percent in rural) than those who did not read books. And secondly, having some playthings also shown to have elevated the development on the track, the percentages were 78.8 percent in urban areas and 74.9 percent in rural areas. Another crucial factor to be noted was the mother's functional difficulties, specifically for children under five years old. In urban areas, around 78 percent of children were shown to be developed on track early if mothers had no functional difficulties. However, the portion was lower in rural areas and was approximately 74 percent.

### 3.3 Collinearity diagnostics

Table 3 illustrated if the independent variables were corresponding or not. Multicollinearity is an increase in interdependence among predictors in a regression model, which can be especially difficult for studies.

**Table 3:** Multicollinearity test among the independent variables.

Variables	Collinearity Statistics	
	Tolerance	VIF
Child Age	0.869	1.151
Child Sex	0.997	1.003
Mother's Education	0.853	1.172
Early Childhood Education	0.838	1.194
Wealth Status	0.840	1.191
Children Nutrition Status	0.967	1.034
Reading Children's Books	0.908	1.101
Having Playthings	0.962	1.040
Mother's Functional Difficulties	0.995	1.005

VIF = Variance Inflation Factor.

Table 3 mainly exhibited the multicollinearity test among independent variables. Every single variable of the current study possessed tolerance (>0.1) and VIF value (<2.5) in terms of cutoff point of Variance Inflation Factor (VIF) and Tolerance limit. Table 3 also recognized that no multicollinearity occurs in this sample, increasing the precision of the estimated coefficient of the regression model of this study.

### 3.4 Identify factors contributing to early child development index

A binary logistic regression model was applied to determine factors related to children's growth in Bangladesh's rural and urban areas and the results was demonstrated in Table 4. The results from Table 4 represented that an increase in children age significantly increased the development index score. Children of 36–47 months old showed a significantly lower odds ratio (OR = 0.57 for urban and OR = 0.56 for rural) than 48–59 months old.

**Table 4:** Binary logistic regression analysis showing the effect of development status among children (36–59 months) by background characteristics in Urban and Rural residence.

Background Characteristics	Early Childhood Development Index			
	Urban		Rural	
	OR (p-value)	CI	OR (p-value)	CI
**Constant**	5.67 (<0.001)	3.37–9.76	4.93 (<0.001)	3.94–6.20
**Child Age**				
36–47 Months	0.57 (<0.001)	0.40–0.67	0.56 (<0.001)	0.49–0.62
48–59 Months (ref.)	1		1	
**Child Sex**				
Male	0.69 (0.002)	0.60–0.97	0.68 (<0.001)	0.62–0.77
Female (ref.)	1		1	
**Mother's Education**				
No Education	0.52 (<0.001)	0.39–0.85	0.72 (<0.001)	0.69–0.96
Primary Education	0.60 (<0.001)	0.46–0.82	0.79 (<0.001)	0.74–0.97
Secondary and above (ref.)	1		1	
**Early Childhood Education**				
Attending	1.99 (<0.001)	1.15–2.28	1.50 (<0.001)	1.33–1.88
Not attending (ref.)	1		1	
**Wealth Status**				
Poor	0.58 (<0.001)	0.50–0.92	0.75 (<0.001)	0.66–0.87
Middle	0.65 (0.02)	0.45–0.91	0.90 (0.20)	0.75–1.04
Rich (ref.)	1		1	
**Children Nutrition Status**				
Severe Malnourished	1.15 (0.71)	0.44–1.92	0.75 (0.02)	0.56–0.89
Moderate Malnourished	1.27 (0.13)	0.95–1.78	0.88 (0.06)	0.79–1.03
Nourished (ref.)	1		1	
**Reading Children's Books**				
≥3 books	1.61 (0.008)	1.01–2.06	1.43 (0.001)	1.13–1.71
<3 books (ref.)	1		1	
**Having Playthings**				
Yes	1.19 (0.46)	0.73–1.81	1.29 (0.004)	1.15–1.59
No (ref.)	1		1	
**Mother's Functional Difficulties**				
Has functional difficulty	1.14 (0.76)	0.51–2.79	0.55 (0.002)	0.43–0.94
Has no functional difficulty (ref.)	1		1	

(ref.) = Reference Category; Statistical Significance: ∗p < 0.05, ∗∗p < 0.01, ∗∗∗p < 0.001.

The gender of a child was a significant indicator of early childhood development. Further investigation showed that male children were more likely to be primitive in at least three domains than female children in urban (31 percent lower) and rural (32 percent lower) areas.

There was a positive association between the mother's educational level and their children's development status under five years old, as observed in Table 4 for both residences. In the urban area, the lower the mother's level of education, the less likely the children in at least three domains (48 percent and 40 percent) will grow up, while the mother's education level was secondary and more. As for the rural residences, when compared between the educational status of mothers, the children with no formal educated mother and primary educated mother were observed to have 28 percent and 21 percent less development respectively than the children with mothers having secondary and above education.

Additionally, this study noted that children attending early education had 1.99 times and 1.50 times higher development on track sequentially compared to the children who did not attend. A positive association was also found between wealth-status and child development index, as for urban areas poor and middle-class household children were 42 percent (OR = 0.52) and 35 percent (OR = 0.65) underdeveloped. For the rural areas, children from poor households had a 25 percent less chance to be developed in at least three domains than the rich household children.

In terms of child nutrition status, it was observed that severely malnourished children experienced 25 percent less development on track in literacy-numeracy, physical, social-emotional, and learning compared to nourished children in rural areas.

Furthermore, it should also be pointed out that reading at least three children's books has greater development opportunities for children in urban and rural areas of Bangladesh. However, no significant association was reported between children due to playthings and early development in urban areas. In rural areas, children who had playthings showed a 1.29 times greater chance to be developed. Also, no significant effect was found on the mother's functional difficulties covariate in the urban section. Mothers with functional difficulties had a 45 percent less chance to develop their children in at least three domains than mothers who had no functional difficulties.

## 4 Discussion

The prime objective was to evaluate early childhood development status in both urban and rural areas of Bangladesh and explore its consequence on various indicators. Our study clarified that participants having children who had developed at least three domains were predominant in Bangladesh.

The findings of this study observed that child age and sex, mother's education, socioeconomic status, child nutrition status, mother's difficulties, having books and playthings were vital determinants of the early child development index for Bangladesh, among them children's demographic features, for instance, age, sex, nutritional status were the main influencing factors.

As proved by this study, children with different demographic characteristics vary in their Early Child Development status. This particular finding was supported by a previous study conducted in Germany in 2011 [25]. Despite having a gender disparity of child development between the age 3–4 years, most researchers observed child development status at the age of 48–59 months. Researchers suggested this age interval for such research because it is the time when a child grows and improves their anger, emotions, and learning domains [26, 27]. An interesting finding of our analysis was that male children were less likely to be developed than female children.

Surprisingly child malnutrition was significantly associated with the early child development status only in rural areas of Bangladesh. Child malnutrition exerts a significant impact on brain growth and development [28]. Children having an excellent nutritional condition (nourished) possessed more ability to be developed. In contrast, malnourished children showed lower ability to do physically and mentally [28, 29, 30]. However, the development index started to be elevated when proper nutritional care was given to nutritionally disadvantaged children. Similar evidence was reported by another study [31].

Mother education had a significant impact on child development in both urban and rural areas of Bangladesh. In the present study, an inverse association was found between child development and mother education. However, according to the viewpoints of [32, 33], it can be easily concluded that there was a significant association between the mother's education level and the early growth of her children. It should be noted, mothers with lower educational status cannot bring up developed children properly. This result was in line with another previous study conducted in Bangladesh and Indonesia [32, 34]. An increasing trend in early childhood development and nutritional status has been observed when maternal education increases [34, 35, 36, 37].

The results indicate that in both urban and rural areas of Bangladesh, those children who attend early school were more likely to have developed than those children who did not attend school early. In urban areas, children who attend school early were more developed than children who reside in rural areas of Bangladesh. Also, children attending early school showed an acceleration in growth than children who did not participate in school early [38, 39, 40].

Our present study also revealed that wealth status was the prime predictor of ECD status in both areas of Bangladesh. There was a positive association between wealth status and child development. Children coming from families with rich households had higher chances of having ECD status than their counterparts. This finding corroborated with other studies conducted in Nepal [41, 42]. A further study also revealed that wealth-status was appreciatively correlated with children's physical and mental growth. At the same time, low-income families were more likely to be experienced a deficit in nutritional status [43].

In addition, this study showed that children who read books had a significant effect on their early development status on children. Furthermore, children who read three or more books experienced an overall increase in development compared to children who do not read less than three books in both areas of Bangladesh. Another separate prior study revealed that the number of books directly correlates positively and independently to ECDI [13, 33]. Nonetheless, these findings made the book a prime component for improving children's brain and cognitive capacity [44].

Playthings always had a dominant role in children's development particularly in the rural areas of Bangladesh. As per our study, having playthings appeared to had strongly associated with the development of children below five years old [45]. This particular result was found to be similar to another analysis carried out in Bangladesh [13]. It was prominent that development was elevated in children exposed to various playthings within 36–59 months old.

Our study showed that mother functional difficulties had a significant impact on child development only in rural areas of Bangladesh. Moreover, mothers with functional difficulties manifested to have poor overall developed children. Although this study didn't notice the effects of some variables, namely, nutrition and playthings in urban areas, still rural areas, showed a more significant impact on such variables.

## 5 Conclusions

Child care is valued vastly in Bangladeshi society. The children development index score aged below five years is not yet satisfactory. In this study, we conclude that a child's age, child sex, mother's education, early childhood education, wealth status, and reading children's books were the significant factors of early child development in both urban and rural areas of Bangladesh. However, children's nutrition status, having playthings, and mother's functional difficulties were significant only in Bangladesh's rural areas. This study strongly disclosure that early childhood development in both places, mainly rural areas, is lower. This research explores that poor people are still present and need to reduce poverty to achieve SDG 1 and increase children literacy-numeracy, physical, social-emotional, and learning domain development, as socio-economic status and childhood development are positively related to each other. Mother's education is also directly involved with under-five childhood development. Unfortunately, the bitter truth is that the education system for girls in Bangladesh is still not up to the mark. However, SDG 4 states that the education system needs to be improved by 2030. We can also conclude that a child's age and sex, child nutrition status are the significant determinants in this study. So, all the significant findings of this study can help the policymaker to improve children's development index at an early age in both areas of Bangladesh. The government should take several actions to enhance education for women and reduce poverty, which is positively associated with early childhood development in both residences of Bangladesh. Public programs should aim to increase significant factors that may increase children's development at age three to below five years.

However, existing practices' virtues could be remarkably reinforced if initiatives were launched to enrich mothers and caregiver's awareness and practices upon various aspects of children's cognitive and emotional development.

## Declarations

### Author contribution statement

Iqramul Haq: Conceived and designed the experiments; Performed the experiments; Contributed reagents, materials, analysis tools or data.

Md. Isamil Hossain: Conceived and designed the experiments; Performed the experiments; Analyzed and interpreted the data; Wrote the paper.

Maliha Afroj Zinnia, Mafruha Sultana Mila, Md. Iqbal Hossain Nayan: Contributed reagents, materials, analysis tools or data; Wrote the paper.

### Funding statement

This research did not receive any specific grant from funding agencies in the public, commercial, or not-for-profit sectors.

### Data availability statement

Data associated with this study is available at the United Nations Children's Fund (UNICEF) under https://mics.unicef.org/surveys.

### Declaration of interests statement

The authors declare no conflict of interest.

### Additional information

No additional information is available for this paper.
